# Tetrakis(2,6-di­methyl­pyridinium) di­hydrogen deca­vanadate dihydrate

**DOI:** 10.1107/S1600536814011118

**Published:** 2014-05-21

**Authors:** Erik Rakovský, Lukáš Krivosudský

**Affiliations:** a Comenius University, Faculty of Natural Sciences, Department of Inorganic Chemistry, Mlynská dolina CH2, 842 15 Bratislava, Slovak Republic

## Abstract

The structure of the title compound, (C_7_H_10_N)_4_[H_2_V_10_O_28_]·2H_2_O, was solved from a non-merohedrally twinned crystal (ratio of twin components ∼0.6:0.4). The asymmetric unit consists of one-half deca­vanadate anion (the other half completed by inversion symmetry), two 2,6-di­methyl­pyridinium cations and one water mol­ecule of crystallization. In the crystal, the components are connected by strong N—H⋯O and O—H⋯O hydrogen bonds, forming a supra­molecular chain along the *b*-axis direction. There are weak C—H⋯O inter­actions between the chains.

## Related literature   

For our previously published research on polyoxidovanadates, see: Rakovský & Gyepes (2006 ▶); Pacigová *et al.* (2007 ▶); Klištincová *et al.* (2008 ▶, 2010 ▶); Bartošová *et al.* (2012 ▶). For more general background to their applications, see: Crans (1998 ▶); Hagrman *et al.* (2001 ▶). Other deca­vanadates with pyridinium derivatives as the cations have been reported by Asgedom *et al.* (1996 ▶); Arrieta *et al.* (1988 ▶); Santi­ago *et al.* (1988 ▶). For IR spectra inter­pretation, see: Ban-Oganowska *et al.* (2002 ▶); Elassal *et al.* (2011 ▶); Medhi & Mukherjee (1965 ▶). For hydrogen-bond criteria, see: Jeffrey (1997 ▶).
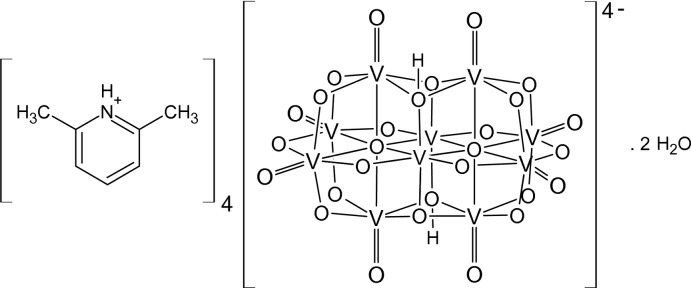


## Experimental   

### 

#### Crystal data  

(C_7_H_10_N)_4_[H_2_V_10_O_28_]·2H_2_O
*M* *_r_* = 1428.09
Monoclinic, 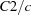

*a* = 24.7777 (5) Å
*b* = 8.35654 (16) Å
*c* = 25.0089 (6) Å
β = 113.878 (3)°
*V* = 4735.0 (2) Å^3^
*Z* = 4
Mo *K*α radiation
μ = 1.98 mm^−1^
*T* = 293 K
0.41 × 0.22 × 0.08 mm

#### Data collection  

Oxford Diffraction Gemini R diffractometer
Absorption correction: gaussian (*CrysAlis PRO*; Agilent, 2014 ▶) *T*_min_ = 0.575, *T*_max_ = 0.873
59285 measured reflections
5867 independent reflections
5086 reflections with *I* > 2σ(*I*)
*R*_int_ = 0.032

#### Refinement  

*R*[*F* ^2^ > 2σ(*F* ^2^)] = 0.031
*wR*(*F* ^2^) = 0.084
*S* = 1.08
5867 reflections
344 parameters
6 restraints
H atoms treated by a mixture of constrained and restrained refinement
Δρ_max_ = 0.77 e Å^−3^
Δρ_min_ = −0.39 e Å^−3^

### 

Data collection: *CrysAlis PRO* (Agilent, 2014 ▶); cell refinement: *CrysAlis PRO*; data reduction: *CrysAlis PRO*; program(s) used to solve structure: *SIR2004* (Burla *et al.*, 2005 ▶); program(s) used to refine structure: *SHELXL2014*/1 (Sheldrick, 2008 ▶); molecular graphics: *DIAMOND* (Brandenburg, 2010 ▶); software used to prepare material for publication: *OLEX2* (Dolomanov *et al.*, 2009 ▶) and *publCIF* (Westrip, 2010 ▶).

## Supplementary Material

Crystal structure: contains datablock(s) I, Ie. DOI: 10.1107/S1600536814011118/gk2606sup1.cif

Structure factors: contains datablock(s) I. DOI: 10.1107/S1600536814011118/gk2606Isup2.hkl

CCDC reference: 1003037

Additional supporting information:  crystallographic information; 3D view; checkCIF report

## Figures and Tables

**Table 1:** Hydrogen-bond geometry (Å, °)

*D*—H⋯*A*	*D*—H	H⋯*A*	*D*⋯*A*	*D*—H⋯*A*
O13—H13⋯O1^i^	0.80 (2)	2.00 (2)	2.789 (2)	172 (3)
N1—H1⋯O9	0.82 (2)	1.81 (2)	2.625 (2)	178 (3)
C15—H15⋯O2^ii^	0.93	2.54	3.396 (3)	152
N2—H2⋯O1*W*	0.83 (2)	1.89 (2)	2.689 (3)	163 (3)
C21—H21*A*⋯O4^iii^	0.96	2.62	3.270 (3)	125
C21—H21*B*⋯O5^iv^	0.96	2.50	3.454 (3)	171
C24—H24⋯O12^v^	0.93	2.49	3.297 (3)	145
C25—H25⋯O7^v^	0.93	2.53	3.237 (3)	134
C25—H25⋯O10^v^	0.93	2.51	3.264 (3)	138
C27—H27*B*⋯O1^i^	0.96	2.46	3.347 (4)	153
O1*W*—H1*W*⋯O11^i^	0.83 (2)	2.02 (2)	2.833 (2)	168 (3)
O1*W*—H2*W*⋯O8	0.83 (2)	1.90 (2)	2.718 (2)	171 (3)

Symmetry codes: (i) 
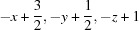
; (ii) 
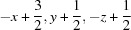
; (iii) 
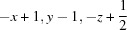
; (iv) 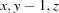; (v) 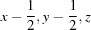.

**Fig. 1.**
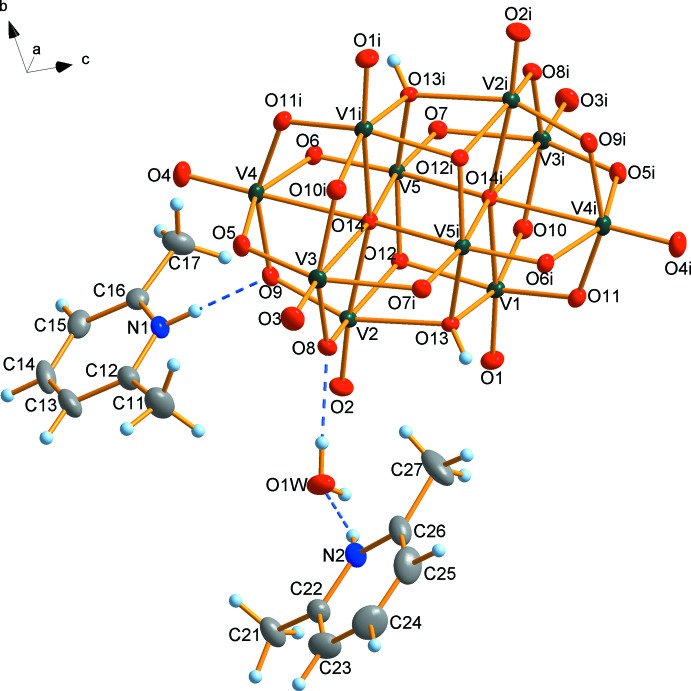
The structure of the title compound, showing the atom labelling scheme and hydrogen bonding interactions (dashed lines). Displacement ellipsoids are drawn ar the 30% probability level. The symmetry operation: (i) 3/2 – x, 3/2 – y, 1 – z.

**Fig. 2.**
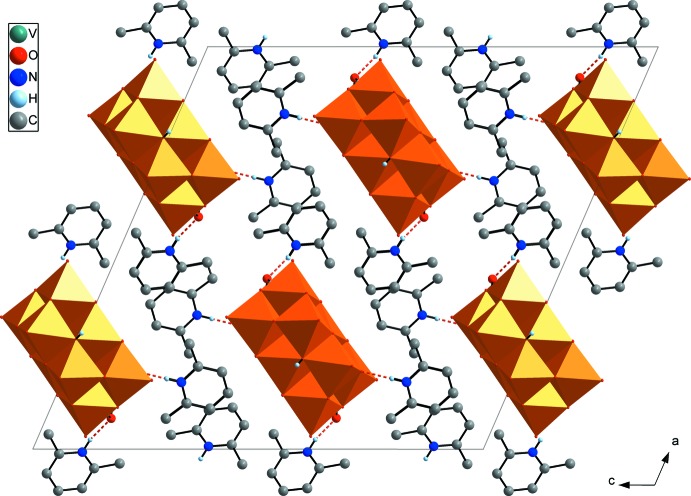
A view of the cell packing of (I) along the b axis. Supramolecular chains are running in the b axis direction, N–H···O and O–H···O hydrogen bonds are drawn as red dashed lines. Carbon-bound hydrogen atoms are omitted for clarity.

## supplementary crystallographic information

### 1. Comment

The reaction system V_2_O_5_ – 2,6-dimethylpyridine – H_2_O – HClO_4_ was studied as a part of our study of the formation of transition metal complexes with substituted pyridinium ligands in the presence of polyoxovanadate anions. We wish to obtain a better understanding of the role of the counter-ion in the formation of H*_n_*V_10_O_28_^(6–^*^n^*^)–^ species and the influence of the cation and the decavanadate anion protonation mode on the IR spectra and information about possible side products of the syntheses. This article is a continuation of our previous work on salts of polyoxovanadates with organic cations (Rakovský & Gyepes, 2006; Pacigová *et al.*, 2007). The oxovanadates(V) and peroxovanadium compounds are also of great interest in biochemistry and medicine because of their diverse biological activities (Crans, 1998). Heterobimetallic compounds containing, beside polyoxovanadate core, entities composed of other transition metals bound to organic ligands have been extensively studied due to their potential applications in the field of catalysis and material science (Hagrman *et al.*, 2001; Klištincová *et al.*, 2008; Klištincová *et al.*, 2010; Bartošová *et al.*, 2012). Several decavanadates with pyridine and its derivatives are already known. Asgedom *et al.* (1996) reported the structure of (C_5_H_6_N)_6_V_10_O_28_.2H_2_O. Pyridinium cations are bonded directly to the decavanadate anions *via* hydrogen bonds as it is in (C_7_H_10_N)_3_H_3_V_10_O_28_.H_2_O (Arrieta *et al.*, 1988) and (C_6_H_8_N)_3_H_3_V_10_O_28_.H_2_O (Santiago *et al.*, 1988).

The system mentioned above was studied in the pH range 2.5–7 and the crystalline product was only obtained at pH 2.5, which is typical pH value for the dihydrogendecavanadate formation. The asymmetric unit of the title compound, (I), consists of one-half decavanadate anion of *C*_i_ symmetry, lying on a special position on the centre of symmetry, which is protonated on the µ-OV_3_ bridging atom O13, two 2,6-dimethylpyridinium cations and one water molecule of crystallization (Fig. 1). The terminal vanadium-oxygen bond lengths are in the range 1.5916 (17)–1.6153 (16) Å, with an average value of 1.601 (10) Å. The bond lengths of the bridging O atoms with coordination numbers two are in the range 1.6768 (14)–2.0752 (15) Å with the mean value of 1.84 (12) Å . There are 2 types of µ-OV_3_ bridging oxygen groups present: unprotonated (O12) with bond lengths in the range 1.8757 (15)–1.9931 (14) Å with the mean value of 1.95 (6) Å, and protonated (O13) with bond lengths in the range 2.0678 (15)–2.1170 (14) Å with the mean value of 2.10 (3) Å. Bond lengths of the six-coordinated µ-OV_6_ oxygen atom (O14) are in the range 2.0592 (13)–2.3588 (14) Å with an average value of 2.24 (13) Å.

The supramolecular structure is formed by *D*–H···O hydrogen bonds [*D* = N, O or C, with H···O ≤ 2.72 Å and *D*–H···O > 120° (Jeffrey, 1997)] between cations and anions, cations and water molecules, between two anions and between water molecules and anions (Table 1). Adjacent [H_2_V_10_O_28_]^4–^ anions are mutually linked together by the system of strong hydrogen bonds: directly by two anion-anion hydrogen bonds O13–H1V···O1 and *via* two bridging water molecules, where water acts as a H-bond donor for both anions, forming O1W–H1W···O11 and O1W–H2W···O8 hydrogen bonds. N–H group of the first cation is donating H-bond to the anion, thus forming N1–H10···O9 hydrogen bond, N–H group of the second cation acts as a H-bond donor for the water molecule, forming N2–H20···O1W hydrogen bond. This system of hydrogen bonds is forming supramolecular chain running in the *b* axis direction (Fig. 2).

The C–H···O weak hydrogen bonds present in the structure are involved in the interaction between neighbouring supramolecular chains, with exception of C21–H21B···O5 and C27–H27B···O1 bonds, which are reinforcing mutual bonding between one of the anions, cation and the water molecule hydrogen-bonded to the cation.

### 2. Experimental

All reactants with the exception of purified vanadium pentoxide were obtained commercially and used without further purification.

Purified vanadium pentoxide was prepared as follows: to 1.5 l of water, NH_4_VO_3_ (50 g) and NH_3_ (60 ml, *w* = 25%) were added. The mixture was stirred and heated in a water bath until the temperature reached 343 K and left cool down for about 1 h. After cooling the mixture was filtered. White NH_4_VO_3_ was precipited by adding of crystalline NH_4_NO_3_ (70 g) to the filtrate, filtered out and washed with distilled water (20 ml) and ethanol (20 ml). The product was dried on air. Purified NH_4_VO_3_ was heated in a porcelain dish at 773 K for at least 2 h.

2 NH_4_VO_3_→ V_2_O_5_ + 2 NH_3_ + H_2_O

Test for purity of prepared V_2_O_5_: small amout of the product added to cold 1 *M* solution of KOH in a test tube completely dissolves.

Synthesis of the (C_7_H_10_N)_4_H_2_V_10_O_28_.2H_2_O (I): V_2_O_5_ (0.9 g, 0.005 mol) was dissolved after stirring overnight in 100 ml of 0.1 *M* solution of 2,6-dimethylpyridine. Yellow solution obtained was filtered and adjusted to pH 2.5 with 4 *M* HClO_4_. Orange plate crystals were isolated after standing 5 days at 277 K.

Vanadium was determined gravimetrically as V_2_O_5_. C, H and N were estimated on a CHN analyser (Carlo Erba). Analysis calculated for C_28_H_46_N_4_O_30_V_10_ (found): C 23.55 (23.59), H 3.25 (3.21), N 3.92 (3.89), V 35.67 (35.58).

The FT—IR spectra were performed with a Nonius 6700 FTIR spectrophotometer in nujol mulls. The IR spectrum of prepared compound exhibits characteristic bands of the decavanadate anion [965 (*s*), 944 (*s*), 926 (*m*), 829 (*s*) and 589 (*m*) cm^-1^] (Klištincová *et al.*, 2010) as well as characteristic bands for protonated 2,6-dimethylpyridine (2534 (*sh*) – ν(NH^+^); 1629 (*s*), 1641 (*s*) – δ(NH^+^)) and other bands for the base (716 (*s*), 794 (*s*) – δ(CH); 971 (*s*) – ν(C–CH_3)_; 1175 (*m*), 1280 (*m*) – δ(CH comb.), 1415 (*m*) – ν(C–C) or ν(C–N)) (Ban-Oganowska *et al.*, 2002; Elassal *et al.*, 2011; Medhi *et al.*, 1965).

### 3. Refinement

The selected crystal was a non-merohedral twin with the twin law: -1 0 0; 0 -1 0; 1 0 1 (given by rows) and the domain volume ratio approx. 0.6:0.4. The structure was solved and refined from detwinned HKLF 4 data, however, due to approximately equal domain volume ratio, some reflections were strongly affected (typically *F*_o_ >> *F*_c_) by twinning; these reflections were omitted in the final stages of the refinement.

The H atoms bound to the C atoms of the cations were placed in geometrically idealized positions (C–H = 0.93 Å) and constrained to ride on their parent atoms [*U*_iso_(H) = 1.2 *U*_eq_(C)] with the exception of the methyl groups, which were treated as rigid rotors [C–H = 0.96 Å, *U*_iso_(H) = 1.5 *U*_eq_(C)]. The H atoms bound to N atoms were refined semi-freely using distance restraint (N–H = 0.86 (2) Å) and with *U*_iso_(H) = 1.5 *U*_eq_(N). The H atoms of the anion and water molecule were located in a difference map and refined with *d*(O–H) = 0.82 (2) Å and *d*(H···H) = 1.36 (2) Å for water molecule.

### Crystal data

**Table:**

(C_7_H_10_N)_4_[H_2_V_10_O_28_]·2H_2_O	*F*(000) = 2848
*M**_r_* = 1428.09	*D*_x_ = 2.003 Mg m^−^^3^
Monoclinic, *C*2/*c*	Mo *K*α radiation, λ = 0.71073 Å
Hall symbol: -C 2yc	Cell parameters from 27323 reflections
*a* = 24.7777 (5) Å	θ = 3.6–28.7°
*b* = 8.35654 (16) Å	µ = 1.98 mm^−^^1^
*c* = 25.0089 (6) Å	*T* = 293 K
β = 113.878 (3)°	Plate, orange
*V* = 4735.0 (2) Å^3^	0.41 × 0.22 × 0.08 mm
*Z* = 4	

### Data collection

**Table:**

Oxford Diffraction Gemini R diffractometer	5867 independent reflections
Radiation source: Enhance (Mo) X-ray Source	5086 reflections with *I* > 2σ(*I*)
Graphite monochromator	*R*_int_ = 0.032
Detector resolution: 10.4340 pixels mm^-1^	θ_max_ = 28.9°, θ_min_ = 3.5°
ω–scans	*h* = −33→33
Absorption correction: gaussian (*CrysAlis PRO*; Agilent, 2014)	*k* = −11→11
*T*_min_ = 0.575, *T*_max_ = 0.873	*l* = −33→33
59285 measured reflections	

### Refinement

**Table:**

Refinement on *F*^2^	Primary atom site location: structure-invariant direct methods
Least-squares matrix: full	Secondary atom site location: difference Fourier map
*R*[*F*^2^ > 2σ(*F*^2^)] = 0.031	Hydrogen site location: difference Fourier map
*wR*(*F*^2^) = 0.084	H atoms treated by a mixture of independent and constrained refinement
*S* = 1.08	*w* = 1/[σ^2^(*F*_o_^2^) + (0.0401*P*)^2^ + 8.437*P*] where *P* = (*F*_o_^2^ + 2*F*_c_^2^)/3
5867 reflections	(Δ/σ)_max_ = 0.001
344 parameters	Δρ_max_ = 0.77 e Å^−^^3^
6 restraints	Δρ_min_ = −0.39 e Å^−^^3^

### Special details

**Table:**

Geometry. All e.s.d.'s (except the e.s.d. in the dihedral angle between two l.s. planes) are estimated using the full covariance matrix. The cell e.s.d.'s are taken into account individually in the estimation of e.s.d.'s in distances, angles and torsion angles; correlations between e.s.d.'s in cell parameters are only used when they are defined by crystal symmetry. An approximate (isotropic) treatment of cell e.s.d.'s is used for estimating e.s.d.'s involving l.s. planes.

### Fractional atomic coordinates and isotropic or equivalent isotropic displacement parameters (Å^2^)

**Table:**

	*x*	*y*	*z*	*U*_iso_*/*U*_eq_	
V1	0.81781 (2)	0.47465 (4)	0.51737 (2)	0.02165 (9)	
V2	0.70304 (2)	0.55586 (4)	0.40183 (2)	0.02228 (9)	
V3	0.60060 (2)	0.74652 (5)	0.41309 (2)	0.02433 (9)	
V4	0.68019 (2)	0.91657 (5)	0.36342 (2)	0.02475 (9)	
V5	0.79281 (2)	0.82080 (4)	0.47073 (2)	0.01834 (8)	
O1	0.82181 (7)	0.30171 (19)	0.49035 (7)	0.0303 (3)	
O2	0.70776 (8)	0.3908 (2)	0.37228 (7)	0.0342 (4)	
O3	0.53181 (7)	0.7150 (2)	0.39512 (8)	0.0374 (4)	
O4	0.67212 (8)	1.0004 (2)	0.30321 (7)	0.0371 (4)	
O5	0.60486 (6)	0.8555 (2)	0.35266 (6)	0.0281 (3)	
O6	0.76907 (6)	0.91441 (18)	0.40574 (6)	0.0237 (3)	
O7	0.86611 (6)	0.84722 (18)	0.50288 (6)	0.0238 (3)	
O8	0.62630 (6)	0.55718 (18)	0.39335 (6)	0.0254 (3)	
O9	0.69435 (7)	0.69902 (19)	0.34630 (6)	0.0258 (3)	
O10	0.88874 (6)	0.55388 (19)	0.54352 (7)	0.0254 (3)	
O11	0.81944 (7)	0.41941 (18)	0.58919 (7)	0.0261 (3)	
O12	0.78823 (6)	0.60759 (17)	0.44582 (6)	0.0204 (3)	
O13	0.72489 (6)	0.48316 (17)	0.48854 (6)	0.0203 (3)	
O14	0.70334 (6)	0.78365 (17)	0.45323 (6)	0.0192 (3)	
H13	0.7096 (11)	0.407 (3)	0.4957 (11)	0.029*	
N1	0.66681 (9)	0.6432 (3)	0.23502 (8)	0.0326 (4)	
H1	0.6763 (13)	0.660 (4)	0.2699 (8)	0.049*	
C11	0.57585 (15)	0.5319 (5)	0.23387 (15)	0.0640 (10)	
H11A	0.5905	0.4366	0.2566	0.096*	
H11B	0.5364	0.5137	0.2055	0.096*	
H11C	0.5758	0.6181	0.2592	0.096*	
C12	0.61462 (12)	0.5743 (3)	0.20330 (11)	0.0395 (6)	
C13	0.60049 (13)	0.5490 (4)	0.14473 (12)	0.0488 (7)	
H13A	0.5644	0.5035	0.1212	0.059*	
C14	0.63984 (13)	0.5913 (4)	0.12099 (11)	0.0498 (8)	
H14	0.6309	0.5702	0.0818	0.060*	
C15	0.69227 (13)	0.6644 (3)	0.15484 (12)	0.0420 (6)	
H15	0.7182	0.6963	0.1385	0.050*	
C16	0.70592 (11)	0.6900 (3)	0.21315 (11)	0.0344 (5)	
C17	0.76196 (14)	0.7645 (4)	0.25454 (15)	0.0542 (8)	
H17A	0.7806	0.6952	0.2876	0.081*	
H17B	0.7535	0.8656	0.2676	0.081*	
H17C	0.7879	0.7806	0.2351	0.081*	
N2	0.49294 (9)	0.1465 (3)	0.38718 (9)	0.0333 (4)	
H2	0.5234 (10)	0.170 (4)	0.3827 (13)	0.050*	
C21	0.46457 (12)	0.0171 (4)	0.29325 (12)	0.0489 (7)	
H21A	0.4366	−0.0615	0.2702	0.073*	
H21B	0.5038	−0.0253	0.3058	0.073*	
H21C	0.4610	0.1115	0.2702	0.073*	
C22	0.45261 (10)	0.0582 (3)	0.34534 (11)	0.0353 (5)	
C23	0.40287 (13)	0.0113 (4)	0.35294 (15)	0.0536 (8)	
H23	0.3737	−0.0482	0.3242	0.064*	
C24	0.39681 (16)	0.0534 (5)	0.40350 (18)	0.0674 (10)	
H24	0.3634	0.0221	0.4091	0.081*	
C25	0.43990 (16)	0.1415 (5)	0.44557 (15)	0.0629 (10)	
H25	0.4361	0.1680	0.4800	0.075*	
C26	0.48857 (13)	0.1904 (4)	0.43713 (12)	0.0453 (7)	
C27	0.53721 (15)	0.2890 (5)	0.47929 (15)	0.0758 (12)	
H27A	0.5409	0.3863	0.4606	0.114*	
H27B	0.5736	0.2303	0.4920	0.114*	
H27C	0.5286	0.3140	0.5125	0.114*	
O1W	0.57679 (8)	0.2656 (2)	0.35592 (9)	0.0425 (4)	
H1W	0.6068 (11)	0.208 (3)	0.3669 (15)	0.064*	
H2W	0.5882 (14)	0.357 (2)	0.3674 (15)	0.064*	

### Atomic displacement parameters (Å^2^)

**Table:**

	*U*^11^	*U*^22^	*U*^33^	*U*^12^	*U*^13^	*U*^23^
V1	0.02131 (17)	0.01978 (18)	0.02536 (18)	0.00097 (13)	0.01099 (14)	0.00127 (13)
V2	0.02531 (18)	0.02300 (18)	0.01948 (17)	−0.00306 (14)	0.01005 (14)	−0.00278 (13)
V3	0.01854 (17)	0.0285 (2)	0.02501 (19)	−0.00254 (13)	0.00786 (14)	0.00077 (14)
V4	0.02526 (18)	0.0288 (2)	0.02013 (18)	−0.00009 (14)	0.00916 (14)	0.00546 (14)
V5	0.01910 (16)	0.01996 (17)	0.01882 (17)	−0.00175 (12)	0.01063 (13)	0.00133 (12)
O1	0.0325 (8)	0.0226 (8)	0.0393 (9)	0.0017 (6)	0.0182 (7)	−0.0010 (7)
O2	0.0425 (9)	0.0288 (9)	0.0352 (9)	−0.0049 (7)	0.0196 (8)	−0.0096 (7)
O3	0.0220 (8)	0.0459 (10)	0.0418 (10)	−0.0054 (7)	0.0104 (7)	0.0006 (8)
O4	0.0378 (9)	0.0459 (10)	0.0265 (8)	−0.0009 (8)	0.0119 (7)	0.0116 (8)
O5	0.0229 (7)	0.0341 (9)	0.0238 (7)	−0.0007 (6)	0.0058 (6)	0.0046 (6)
O6	0.0256 (7)	0.0263 (8)	0.0223 (7)	−0.0023 (6)	0.0130 (6)	0.0047 (6)
O7	0.0211 (7)	0.0271 (8)	0.0261 (7)	−0.0026 (6)	0.0125 (6)	−0.0002 (6)
O8	0.0244 (7)	0.0260 (8)	0.0249 (7)	−0.0059 (6)	0.0089 (6)	−0.0028 (6)
O9	0.0289 (8)	0.0316 (8)	0.0180 (7)	−0.0034 (6)	0.0107 (6)	−0.0008 (6)
O10	0.0208 (7)	0.0278 (8)	0.0297 (8)	0.0021 (6)	0.0122 (6)	0.0019 (6)
O11	0.0263 (7)	0.0243 (8)	0.0279 (8)	0.0011 (6)	0.0113 (6)	0.0064 (6)
O12	0.0230 (7)	0.0214 (7)	0.0203 (7)	−0.0012 (5)	0.0125 (5)	−0.0010 (5)
O13	0.0226 (7)	0.0181 (7)	0.0225 (7)	−0.0039 (5)	0.0117 (6)	−0.0003 (5)
O14	0.0201 (6)	0.0216 (7)	0.0179 (6)	−0.0006 (5)	0.0097 (5)	0.0013 (5)
N1	0.0405 (11)	0.0356 (11)	0.0211 (9)	0.0010 (9)	0.0118 (8)	−0.0025 (8)
C11	0.0545 (19)	0.087 (3)	0.0526 (19)	−0.0222 (18)	0.0243 (15)	−0.0066 (18)
C12	0.0385 (13)	0.0433 (15)	0.0332 (13)	−0.0004 (11)	0.0109 (10)	−0.0037 (11)
C13	0.0405 (15)	0.063 (2)	0.0323 (14)	0.0043 (13)	0.0034 (11)	−0.0131 (13)
C14	0.0530 (16)	0.069 (2)	0.0234 (12)	0.0252 (15)	0.0117 (11)	−0.0029 (12)
C15	0.0517 (16)	0.0445 (15)	0.0386 (14)	0.0181 (12)	0.0275 (12)	0.0038 (12)
C16	0.0406 (13)	0.0294 (12)	0.0362 (13)	0.0050 (10)	0.0186 (10)	−0.0030 (10)
C17	0.0502 (17)	0.0553 (19)	0.063 (2)	−0.0145 (14)	0.0293 (15)	−0.0199 (16)
N2	0.0273 (10)	0.0437 (12)	0.0305 (10)	0.0029 (9)	0.0134 (8)	−0.0008 (9)
C21	0.0386 (14)	0.075 (2)	0.0339 (14)	−0.0052 (14)	0.0153 (11)	−0.0131 (14)
C22	0.0291 (11)	0.0425 (14)	0.0365 (13)	−0.0005 (10)	0.0155 (10)	−0.0022 (11)
C23	0.0396 (15)	0.0593 (19)	0.070 (2)	−0.0120 (14)	0.0303 (15)	−0.0107 (16)
C24	0.057 (2)	0.083 (3)	0.087 (3)	−0.0003 (19)	0.056 (2)	0.004 (2)
C25	0.069 (2)	0.087 (3)	0.0500 (18)	0.023 (2)	0.0415 (17)	0.0048 (18)
C26	0.0453 (15)	0.0560 (18)	0.0327 (13)	0.0176 (13)	0.0139 (11)	−0.0036 (12)
C27	0.057 (2)	0.100 (3)	0.053 (2)	0.016 (2)	0.0038 (16)	−0.038 (2)
O1W	0.0294 (9)	0.0320 (10)	0.0617 (13)	−0.0050 (7)	0.0139 (9)	−0.0002 (9)

### Geometric parameters (Å, º)

**Table:**

V1—O1	1.6153 (16)	C11—H11B	0.9600
V1—O10	1.7390 (15)	C11—H11C	0.9600
V1—O11	1.8391 (15)	C11—C12	1.492 (4)
V1—O12	1.9776 (14)	C12—C13	1.378 (4)
V1—O13	2.1170 (14)	C13—H13A	0.9300
V1—O14^i^	2.2818 (14)	C13—C14	1.377 (4)
V2—O2	1.5916 (17)	C14—H14	0.9300
V2—O8	1.8261 (15)	C14—C15	1.374 (4)
V2—O9	1.7788 (15)	C15—H15	0.9300
V2—O12	1.9931 (14)	C15—C16	1.374 (3)
V2—O13	2.1009 (15)	C16—C17	1.491 (4)
V2—O14	2.2952 (14)	C17—H17A	0.9600
V3—O3	1.5990 (16)	C17—H17B	0.9600
V3—O5	1.8035 (16)	C17—H17C	0.9600
V3—O7^i^	2.0752 (15)	N2—H2	0.830 (17)
V3—O8	1.8458 (16)	N2—C22	1.338 (3)
V3—O10^i^	1.9487 (16)	N2—C26	1.348 (3)
V3—O14	2.3486 (14)	C21—H21A	0.9600
V4—O4	1.5983 (16)	C21—H21B	0.9600
V4—O5	1.8468 (15)	C21—H21C	0.9600
V4—O6	2.0208 (15)	C21—C22	1.488 (3)
V4—O9	1.9321 (16)	C22—C23	1.378 (4)
V4—O11^i^	1.8097 (16)	C23—H23	0.9300
V4—O14	2.3588 (14)	C23—C24	1.378 (5)
V5—O6	1.6810 (14)	C24—H24	0.9300
V5—O7	1.6768 (14)	C24—C25	1.371 (5)
V5—O12	1.8757 (15)	C25—H25	0.9300
V5—O13^i^	2.0678 (15)	C25—C26	1.368 (5)
V5—O14^i^	2.0592 (13)	C26—C27	1.488 (5)
V5—O14	2.1015 (13)	C27—H27A	0.9600
O13—H13	0.796 (17)	C27—H27B	0.9600
N1—H1	0.817 (17)	C27—H27C	0.9600
N1—C12	1.343 (3)	O1W—H1W	0.832 (17)
N1—C16	1.349 (3)	O1W—H2W	0.825 (17)
C11—H11A	0.9600		
			
O1—V1—O10	105.91 (8)	V5—O12—V2	107.31 (7)
O1—V1—O11	101.72 (8)	V1—O13—H13	116.6 (19)
O1—V1—O12	100.78 (7)	V2—O13—V1	98.72 (6)
O1—V1—O13	97.47 (7)	V2—O13—H13	121.1 (19)
O1—V1—O14^i^	171.01 (7)	V5^i^—O13—V1	106.09 (6)
O10—V1—O11	96.25 (7)	V5^i^—O13—V2	105.18 (6)
O10—V1—O12	94.27 (7)	V5^i^—O13—H13	107.7 (19)
O10—V1—O13	155.43 (7)	V1^i^—O14—V2	164.90 (7)
O10—V1—O14^i^	82.58 (6)	V1^i^—O14—V3	84.49 (5)
O11—V1—O12	151.43 (6)	V1^i^—O14—V4	83.74 (5)
O11—V1—O13	86.10 (6)	V2—O14—V3	83.90 (5)
O11—V1—O14^i^	79.89 (6)	V2—O14—V4	85.00 (5)
O12—V1—O13	73.70 (6)	V3—O14—V4	81.46 (4)
O12—V1—O14^i^	75.23 (5)	V5—O14—V1^i^	99.36 (6)
O13—V1—O14^i^	73.74 (5)	V5^i^—O14—V1^i^	90.48 (5)
O2—V2—O8	102.71 (8)	V5^i^—O14—V2	98.86 (6)
O2—V2—O9	103.22 (8)	V5—O14—V2	90.18 (5)
O2—V2—O12	100.59 (8)	V5—O14—V3	167.97 (7)
O2—V2—O13	101.07 (8)	V5^i^—O14—V3	88.61 (5)
O2—V2—O14	174.13 (8)	V5—O14—V4	87.61 (5)
O8—V2—O12	152.03 (6)	V5^i^—O14—V4	168.93 (7)
O8—V2—O13	86.72 (6)	V5^i^—O14—V5	102.69 (6)
O8—V2—O14	80.05 (6)	C12—N1—H1	120 (2)
O9—V2—O8	96.54 (7)	C12—N1—C16	124.2 (2)
O9—V2—O12	92.96 (6)	C16—N1—H1	116 (2)
O9—V2—O13	154.09 (7)	H11A—C11—H11B	109.5
O9—V2—O14	81.46 (6)	H11A—C11—H11C	109.5
O12—V2—O13	73.75 (6)	H11B—C11—H11C	109.5
O12—V2—O14	75.43 (5)	C12—C11—H11A	109.5
O13—V2—O14	73.78 (5)	C12—C11—H11B	109.5
O3—V3—O5	105.36 (8)	C12—C11—H11C	109.5
O3—V3—O7^i^	99.44 (8)	N1—C12—C11	117.7 (2)
O3—V3—O8	103.16 (8)	N1—C12—C13	117.6 (3)
O3—V3—O10^i^	100.73 (8)	C13—C12—C11	124.7 (3)
O3—V3—O14	171.75 (7)	C12—C13—H13A	120.0
O5—V3—O7^i^	154.82 (6)	C14—C13—C12	120.0 (3)
O5—V3—O8	93.73 (7)	C14—C13—H13A	120.0
O5—V3—O10^i^	89.65 (7)	C13—C14—H14	119.8
O5—V3—O14	82.56 (6)	C15—C14—C13	120.4 (2)
O7^i^—V3—O14	72.50 (5)	C15—C14—H14	119.8
O8—V3—O7^i^	84.75 (6)	C14—C15—H15	120.4
O8—V3—O10^i^	154.02 (6)	C16—C15—C14	119.2 (3)
O8—V3—O14	78.23 (6)	C16—C15—H15	120.4
O10^i^—V3—O7^i^	81.40 (6)	N1—C16—C15	118.5 (2)
O10^i^—V3—O14	76.69 (6)	N1—C16—C17	117.3 (2)
O4—V4—O5	104.49 (8)	C15—C16—C17	124.2 (3)
O4—V4—O6	101.04 (8)	C16—C17—H17A	109.5
O4—V4—O9	99.69 (8)	C16—C17—H17B	109.5
O4—V4—O11^i^	104.52 (9)	C16—C17—H17C	109.5
O4—V4—O14	173.18 (8)	H17A—C17—H17B	109.5
O5—V4—O6	153.74 (6)	H17A—C17—H17C	109.5
O5—V4—O9	88.34 (7)	H17B—C17—H17C	109.5
O5—V4—O14	81.40 (6)	C22—N2—H2	117 (2)
O6—V4—O14	72.76 (5)	C22—N2—C26	124.0 (2)
O9—V4—O6	81.40 (6)	C26—N2—H2	119 (2)
O9—V4—O14	76.82 (6)	H21A—C21—H21B	109.5
O11^i^—V4—O5	92.39 (7)	H21A—C21—H21C	109.5
O11^i^—V4—O6	87.03 (7)	H21B—C21—H21C	109.5
O11^i^—V4—O9	154.77 (7)	C22—C21—H21A	109.5
O11^i^—V4—O14	78.36 (6)	C22—C21—H21B	109.5
O6—V5—O12	99.81 (7)	C22—C21—H21C	109.5
O6—V5—O13^i^	92.68 (7)	N2—C22—C21	117.5 (2)
O6—V5—O14^i^	163.23 (6)	N2—C22—C23	118.4 (2)
O6—V5—O14	86.60 (6)	C23—C22—C21	124.1 (3)
O7—V5—O6	106.83 (7)	C22—C23—H23	120.3
O7—V5—O12	101.10 (7)	C22—C23—C24	119.3 (3)
O7—V5—O13^i^	93.67 (7)	C24—C23—H23	120.3
O7—V5—O14^i^	88.66 (6)	C23—C24—H24	120.0
O7—V5—O14	164.97 (6)	C25—C24—C23	120.1 (3)
O12—V5—O13^i^	156.90 (6)	C25—C24—H24	120.0
O12—V5—O14^i^	83.00 (6)	C24—C25—H25	119.9
O12—V5—O14	82.74 (6)	C26—C25—C24	120.2 (3)
O13^i^—V5—O14	78.66 (6)	C26—C25—H25	119.9
O14^i^—V5—O13^i^	79.67 (6)	N2—C26—C25	117.9 (3)
O14^i^—V5—O14	77.31 (6)	N2—C26—C27	117.5 (3)
V3—O5—V4	114.58 (8)	C25—C26—C27	124.6 (3)
V5—O6—V4	113.02 (7)	C26—C27—H27A	109.5
V5—O7—V3^i^	110.20 (7)	C26—C27—H27B	109.5
V2—O8—V3	115.45 (8)	C26—C27—H27C	109.5
V2—O9—V4	115.80 (8)	H27A—C27—H27B	109.5
V1—O10—V3^i^	115.08 (8)	H27A—C27—H27C	109.5
V4^i^—O11—V1	116.22 (8)	H27B—C27—H27C	109.5
V1—O12—V2	107.42 (7)	H1W—O1W—H2W	107 (2)
V5—O12—V1	106.42 (7)		

Symmetry code: (i) −*x*+3/2, −*y*+3/2, −*z*+1.

### Hydrogen-bond geometry (Å, º)

**Table:**

*D*—H···*A*	*D*—H	H···*A*	*D*···*A*	*D*—H···*A*
O13—H13···O1^ii^	0.80 (2)	2.00 (2)	2.789 (2)	172 (3)
N1—H1···O9	0.82 (2)	1.81 (2)	2.625 (2)	178 (3)
C15—H15···O2^iii^	0.93	2.54	3.396 (3)	152
N2—H2···O1*W*	0.83 (2)	1.89 (2)	2.689 (3)	163 (3)
C21—H21*A*···O4^iv^	0.96	2.62	3.270 (3)	125
C21—H21*B*···O5^v^	0.96	2.50	3.454 (3)	171
C24—H24···O12^vi^	0.93	2.49	3.297 (3)	145
C25—H25···O7^vi^	0.93	2.53	3.237 (3)	134
C25—H25···O10^vi^	0.93	2.51	3.264 (3)	138
C27—H27*B*···O1^ii^	0.96	2.46	3.347 (4)	153
O1*W*—H1*W*···O11^ii^	0.83 (2)	2.02 (2)	2.833 (2)	168 (3)
O1*W*—H2*W*···O8	0.83 (2)	1.90 (2)	2.718 (2)	171 (3)

Symmetry codes: (ii) −*x*+3/2, −*y*+1/2, −*z*+1; (iii) −*x*+3/2, *y*+1/2, −*z*+1/2; (iv) −*x*+1, *y*−1, −*z*+1/2; (v) *x*, *y*−1, *z*; (vi) *x*−1/2, *y*−1/2, *z*.
